# Follicular GH and IGF1 Levels Are Associated With Oocyte Cohort Quality: A Pilot Study

**DOI:** 10.3389/fendo.2021.793621

**Published:** 2021-12-01

**Authors:** Florence Scheffler, Albane Vandecandelaere, Marion Soyez, Dorian Bosquet, Elodie Lefranc, Henri Copin, Aviva Devaux, Moncef Benkhalifa, Rosalie Cabry, Rachel Desailloud

**Affiliations:** ^1^ Reproductive Medicine and Biology Department and CECOS of Picardy, Amiens University Hospital, Amiens, France; ^2^ Peritox UMR_I 01, CURS, Jules Verne University of Picardy, Amiens, France; ^3^ Endocrine and Bone Biology Department, Amiens University Hospital, Amiens, France; ^4^ Endocrinology, Diabetes, and Nutrition Department, Amiens University Hospital, Amiens, France

**Keywords:** oocyte morphological abnormality, follicular fluid, intracytoplasmic sperm injection, growth hormone, insulin-like growth factor 1, thyroid-stimulating hormone, thyroid hormones, 25-hydroxy vitamin D

## Abstract

**Introduction:**

Oocyte quality contributes to the development of an optimal embryo and thus a successful pregnancy. The objective of this study was to analyse the association between oocyte cohort quality and the follicular levels of growth hormone (GH), insulin-like growth factor 1 (IGF1), 25-hydroxy vitamin D (25OHD), thyroid-stimulating hormone (TSH), free triiodothyronine (fT3), free thyroxine (fT4) and antithyroid antibodies, as a function of intracytoplasmic sperm injection (ICSI) outcomes.

**Material and methods:**

We conducted a prospective comparative pilot study from January 2013 to December 2017. 59 ICSI cycles constituted an abnormal oocyte cohort (n=34 cycles, in which more than 50% of oocytes presented at least one morphological abnormality) and a normal oocyte cohort (n=25 cycles, in which 50% or less of the oocytes presented at least one morphological abnormality). GH, IGF1, 25OHD, TSH, fT3, fT4 and antithyroid antibodies were measured in follicular fluid.

**Results:**

The fertilisation rate was lower in the abnormal oocyte cohort (65.5% *vs.* 80%, respectively, p=0.012). Oocytes’ proportion with at least one abnormality was 79.4% in the abnormal oocyte cohort and 29.0% in the normal oocyte cohort. The mean number of morphological abnormalities per oocyte was significantly higher in the abnormal oocyte cohort. The follicular levels of GH (4.98 *vs.* 2.75 mIU/L, respectively; p <0.01) and IGF1 (72.1 *vs.* 54.2 ng/mL, respectively; p=0.05) were higher in the normal oocyte cohort. There was no association with follicular levels of TSH, fT3, fT4, antithyroid antibodies, or 25OHD.

**Conclusion:**

Oocyte cohort quality appears to be associated with follicular levels of GH and IGF1.

## Introduction

Gamete quality is one of the many factors involved in the success or failure of *in vitro* fertilisation (IVF). With the development of the intracytoplasmic sperm injection (ICSI), a decoronized oocyte’s nuclear maturity and morphological structure can be assessed precisely. Oocyte quality contributes to the development of an optimal embryo and thus a successful pregnancy (1). However, 10 to 60% of the oocytes obtained after controlled ovarian stimulation (COS) for IVF present morphological abnormalities, such as diffuse cytoplasmic granularity, refractile bodies, vacuoles, large perivitelline space, perivitelline debris, irregular shape, and a fragmented or large first polar body (1–5). These morphological abnormalities are not well understood but may be caused by intrinsic factors (such as age and genetic defects) and/or extrinsic factors (such as the stimulation protocol, oocyte culture conditions, and nutrition) (1). Follicular fluid (FF) provides the microenvironment for oocyte maturation (6). It contains hormones with pleiomorphic effects involved in ovarian folliculogenesis, oogenesis, and steroidogenesis. Various studies have shown that growth hormone (GH), insulin-like growth factor 1 (IGF1), thyroid-stimulating hormone (TSH), and thyroid hormones [THs, e.g. free triiodothyronine (fT3) and free thyroxine (fT4)] have an influence on ovarian function. GH has both direct and indirect (IGF1-mediated) stimulatory effects on folliculogenesis, oocyte maturation, and steroidogenesis (7, 8). TH improves granulosa cell proliferation (9), inhibits apoptosis of the latter (10), and contributes to steroidogenesis by increasing the secretion of oestradiol and progesterone by granulosa cells (11, 12). More recently, it was reported that 1-25-hydroxy vitamin D (1-25OHD) is a factor in ovarian folliculogenesis (13, 14) and steroidogenesis (15).

The objective of the present study was to assess the putative association between oocyte cohort quality in an ICSI programme and follicular levels of GH, IGF1, 25-hydroxy vitamin D (25OHD), TSH, fT3, fT4, anti-thyroperoxidase (TPO) antibodies, and anti-thyroglobulin (TG) antibodies, as a function of the ICSI outcomes.

## Materials and Methods

We conducted a prospective pilot study at a reproductive medicine centre at Amiens-Picardie University Hospital (Amiens, France) from January 2013 to December 2017. The study protocol was approved by the local investigational review board (Amiens, France; reference: RCB 2011-A00634-37). All the study participants (couples participating in an ICSI programme, regardless of the indication) provided their informed consent. All the women were euthyroid at the time when their ICSI programme started. The main inclusion criteria were first or second ICSI cycle, age under 36 (for women) or 45 (for men), and a sperm concentration greater than 5x10^6^/mL. Patients with stage III/IV endometriosis and/or ovarian endometrioma were excluded. We also excluded ICSI cycles with less than 4 mature oocytes after decoronization.

### COS and IVF Protocols

Two COS protocols were used: a gonadotropin-releasing hormone (GnRH) long agonist protocol and a GnRH antagonist protocol.

The long agonist protocol involved pituitary downregulation with a GnRH agonist (triptorelin acetate: Décapeptyl^®^, Ipsen Pharma, France; 0.1 mg per day for 14 days, starting in the midluteal phase), followed by the administration of recombinant human follicle-stimulating hormone (rFSH: Puregon^®^, Organon, France, or Gonal-F^®^, Merck Serono SAS, France) or human menopausal gonadotropin (HMG, Menopur^®^, Ferring, France), in combination with a GnRH agonist (triptorelin acetate: Décapeptyl^®^, Ipsen Pharma, France; 0.05 mg per day).

In the antagonist protocol, rFSH was administered subcutaneously each day from day 2 of the cycle until a 14 mm dominant follicle was detected. Cetrorelix acetate (Cetrotide^®^, Merck Serono, France; 0.25 mg per day) was then administered daily until the recombinant human chorionic gonadotropin (rhCG) day (Ovitrelle^®^, Merck Serono SAS).

The stimulation protocols and the type and dose of FSH were chosen by the gynaecologist, as a function of the patient’s age, body mass index (BMI), and ovarian reserve (anti-Müllerian hormone (AMH) level, antral follicle count, and basal FSH level).

Patients were monitored clinically using transvaginal pelvic ultrasound and assays for oestradiol, progesterone, and luteinizing hormone. The rFSH/HMG dose level was adjusted according to the follicular growth measured during the monitoring phase. When at least three follicles had reached a diameter of more than 16 mm, a 250 μg dose of rhCG was administered. Oocytes were retrieved 36 h after hCG administration, *via* ultrasound-guided transvaginal follicular aspiration.

Cumulus cells were mechanically and enzymatically decoronized from the oocyte complexes 38 h after the rhCG administration. All mature oocytes were used for ICSI according to standard protocols regardless of their morphology, and fertilisation was assessed 16–18 hours after sperm injection. The morphology was assessed according to the Istanbul consensus criteria [for day 2/3 embryos (16)] or Gardner’s criteria [for blastocysts (17)]. Progestin (Utrogestan^®^ 400 mg, Besins International, France) was used for luteal support. Pregnancy was defined as a serum hCG level >100 IU/L 14 days after embryo transfer.

### Group Formation

**Figure 1:**
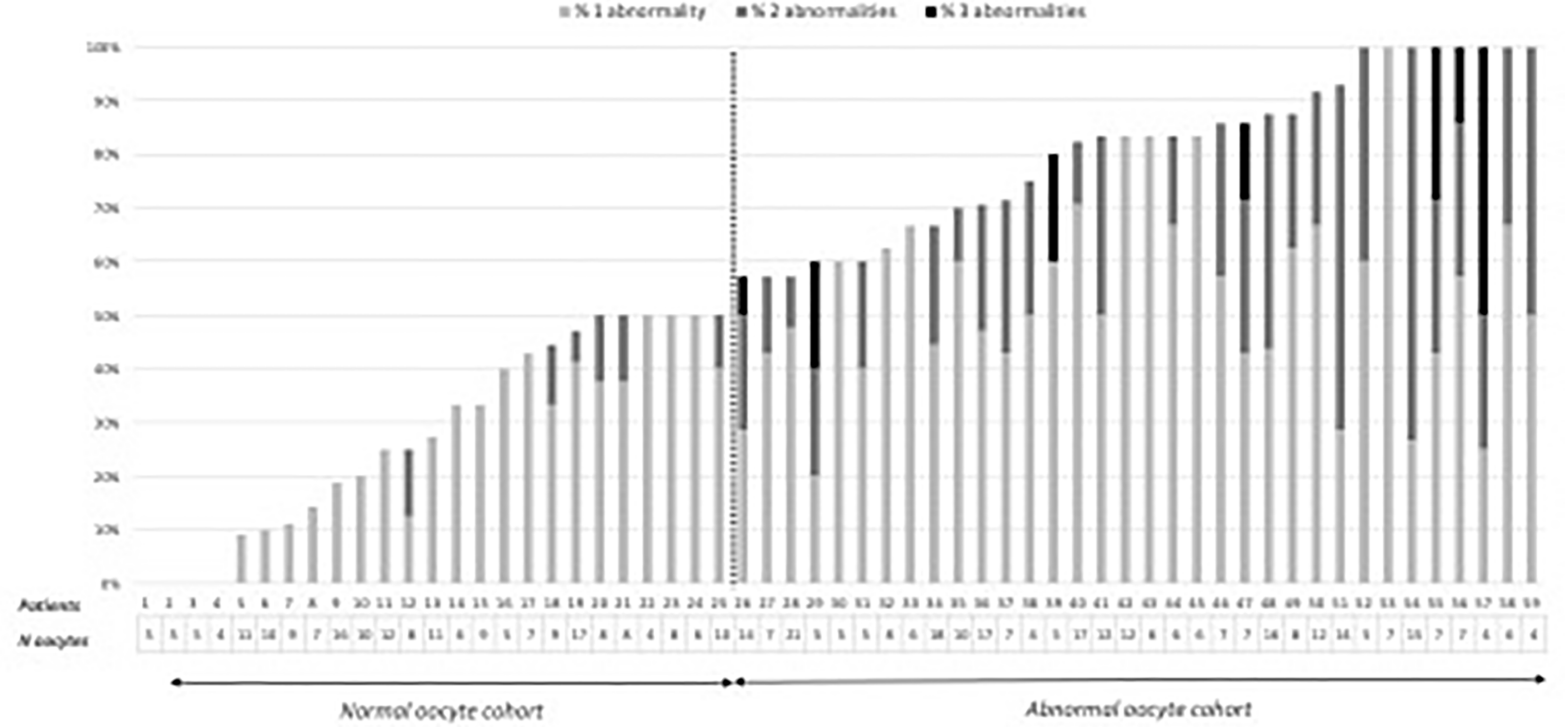
Distribution of the number of oocytes and oocyte abnormalities by patient in the oocyte cohorts.

After decoronization, oocyte morphology was evaluated under an inverted microscope (Nikon^®^ 2000TU, France) equipped with Hoffman modulation contrast optics. The following oocyte morphological abnormalities were assessed and counted: diffuse cytoplasmic granularity, refractile bodies, vacuoles, sticky, or soft oocytes, large perivitelline space, and perivitelline debris. Depending on the proportion of their oocytes with one or more morphological abnormalities, patients were classified into an abnormal oocyte cohort (with more than 50% of the oocytes presenting at least one abnormality) and a normal oocyte cohort (with 50% or less of oocytes presenting at least one abnormality). The 50% cut-off was chosen following the analysis of the distribution of oocyte abnormalities in the total cohort (**Figure 1**).

### Preparation of Follicular Fluid Samples

Following oocyte decoronization, each patient’s remaining FF samples were pooled. After the removal of cells by centrifugation at 2000 g for 10 minutes, the supernatant was recovered, stored at -20°C, and thawed immediately prior to analysis. FF samples that were contaminated with blood were excluded.

### Hormone and Antithyroid Antibody Assays

All hormone assays and antithyroid antibody screens were carried out as a single series at the end of the study. ELISAs were used to determine levels of TSH, fT3, fT4 (ELISA VISTA 500™, Siemens Healthcare Diagnostics, Germany), 25OHD, anti-TPO antibodies (CENTAUR XP™, Siemens Healthcare Diagnostic, Germany), GH and IGF1 (IMMULITE 2000XPi, Siemens Healthcare Diagnostic, Germany). Anti-TG antibody titres were measured using an immunoradiometric assay (Immunotech^®^, Beckman Coulter, Czech Republic). The intra- and inter-assay coefficients of variation were below 10% in all assays.

### Statistical Analysis

All statistical analyses were performed with *pvalue.io* software (18). Data were expressed as the median [interquartile range] or the frequency (percentage). Intergroup differences groups were probed with a Mann-Whitney test (for quantitative variables) or a chi-squared test or Fisher’s exact test (for qualitative variables). The threshold for statistical significance was set to p<0.05. As this pilot study was exploratory, no calculation of the sample size was carried out.

## Results

### Characteristics of the Study Population and ICSI Outcomes

71 ICSI cycles were included in the study. 4 endometriotic patients were excluded as well as 8 ICSI with less than 4 mature oocytes. 59 ICSI were analysed in total. There were no significant differences between the normal oocyte cohort (n=34 cycles) and the abnormal oocyte cohort (n=25 cycles) with regard to age, BMI, current smoking status, ovarian reserve, and duration of infertility (**Table 1**).

**Table 1:** Characteristics of the study population, as a function of the oocyte cohort quality.

	Abnormal oocyte cohort (n = 34 cycles)	Normal oocyte cohort (n = 25 cycles)	p
**Duration of infertility (years)**	3.50 [3.00; 5.00]	4.00 [3.00; 4.00]	0.85
**Characteristics of the women:**			
*- Age (years)*	31.5 [29.0; 34.8]	30.0 [29.0; 34.0]	0.69
*- BMI (kg/m2)*	23.0 [21.2; 27.0]	21.0 [20.0; 25.0]	0.096
*- Current smoker*	15%	20%	0.73
*- POF*	5.9%	8%	1
*- PCOS*	35%	24%	0.35
*- FSH on day 3 (IU/L)*	6.05 [4.82; 6.95]	5.60 [4.40; 7.00]	0.7
*- AMH (ng/mL)*	3.7 [2.8; 8.60]	3.30 [2.00; 4.50]	0.4
**Characteristics of the men’s sperm**			
***-*** *Sperm concentration (M/ml)*	38.2 [16.3; 79.3]	22.9 [12.0; 59.2]	0.19
*- Progressive motility (%)*	35.0 [30.0; 45.0]	27.5 [15.0; 40.0]	0.07

The data are expressed as the median [interquartile range] or the frequency (percentage).

BMI, body mass index; POF, premature ovarian failure; PCOS, polycystic ovary syndrome; FSH, follicle-stimulating hormone; AMH, anti-Müllerian hormone.

There were no differences between the normal and abnormal oocyte cohorts with regard to the ovarian response, ovarian stimulation characteristics, and ICSI outcomes (**Table 2**). The fertilisation rate was significantly lower in the abnormal oocyte cohort than in the normal oocyte cohort (65.5% *vs.* 80%, respectively, p=0.012). There were no intergroup differences in the embryonic development rate or the ICSI outcomes.

**Table 2:** The ovarian response, oocyte characteristics, and ICSI outcomes as a function of the oocyte cohort quality.

	Abnormal oocyte cohort (n = 34 cycles)	Normal oocyte cohort (n = 25 cycles)	p
**COS parameters**			
**Ovarian stimulation protocol:**			
*- GnRH antagonist*	9%91%	12%88%
*- Long GnRH agonist*	91%	88%	0.69
- *Duration of COS (day)*	11.5 [10.2; 13.0]	12.0 [10.0; 13.0]	0.66
- *Total dose of rFSH/HMG (IU)*	1775 [1350; 2681]	1650 [1350; 2200]	0.67
- *E2 level on the hCG day (pg/ml)*	2127 [1585; 2828]	2003 [1450; 2735]	0.94
**Oocyte characteristics**			
- *Number of oocytes retrieved*	14.5 [9.25; 19.0]	12.0 [8.00; 15.0]	0.19
- *Number of oocytes injected*	7.00 [6.00; 12.0]	8.00 [6.00; 10.0]	0.98
- *Proportion of matures oocytes*	73.7 [59.5; 77.8]	75.0 [68.8; 83.3]	0.15
- *Proportion of abnormal oocytesNumber of morphological abnormalities per oocyte:*	83.3 [67.5; 92.6]	27.3 [11.1; 47.1]	**<0.001**
*- One abnormality*	52.8%	25.7%	**<0.001**
*- Two abnormalities*	23.9%	2.9%	
*- Three or more abnormalities*	3.2%	0%	
**ICSI outcomes**			
- *Fertilization rate*	65.5 [44.7; 83.3]	80.0 [68.8; 88.9]	**0.012**
- *“Day 2 embryo” development rate*	50.0 [25.0; 88.9]	62.5 [39.6; 77.5]	0.66
- *FET rate*	65%	72%	0.55
- *Embryo cryopreservation rate*	44%	40%	0.75
- *Pregnancy rate*	45%	50%	0.77
- *Live birth rate*	32%	44%	0.41

The data are expressed as the median [interquartile range] or the frequency (percentage).

GnRH, gonadotropin-releasing hormone; COS, control ovarian stimulation; rFSH, recombinant follicle-stimulating hormone; HMG, human menopausal gonadotropin; E2, oestradiol; hCG, human chorionic gonadotropin; FET, fresh embryo transfer.

### Distribution of Oocyte Morphological Abnormalities

**Figure 2:**
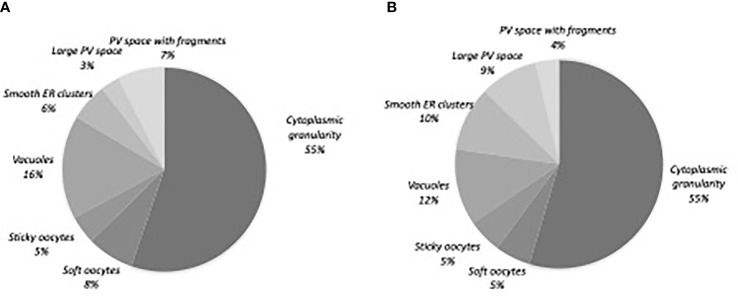
Distribution of the oocyte morphological abnormalities. **(A)** Normal oocyte cohort, n=61 oocytes **(B)** Abnormal oocyte cohort, n=246 oocytes. PV, perivitelline; ER, endoplasmic reticulum.

Of the 520 matures oocytes analysed, 307 (59%) presented one or more morphological abnormality. This proportion was 79.4% (n= 246) in the abnormal oocyte cohort and 29.0% (n=61) in the normal oocyte cohort. 217 oocytes (41.7%) had one abnormality, 80 (15.4%) had two, and 10 (1.9%) had three or more (**Table 2**). Diffuse cytoplasmic granularity accounted for more than half the abnormalities (**Figure 2**).

### Hormonal and Antithyroid Antibodies Assay in FF

The follicular GH level was significantly higher in the normal oocyte cohort than in the abnormal oocyte cohort (4.98 *vs.* 2.75 mIU/L, respectively; p <0.01); the same was true for follicular levels of IGF1 (72.1 *vs.* 54.2 ng/mL, respectively; p=0.05) (**Table 3**). The intergroup differences in follicular levels of TSH, fT3, fT4, 25OHD and antithyroid antibodies were not statistically significant.

**Table 3:** Follicular hormone levels and prevalence of antithyroid antibodies in the FF, as a function of oocyte cohort quality.

	Abnormal oocyte cohort (n = 34 cycles)	Normal oocyte cohort (n = 25 cycles)	**p**
**Somatotropic axis:**			
* - GH (mIU/L)*	2.75 [1.95; 4.24]	4.98 [2.84; 7.05]	**<0.01**
* - IGF1 (ng/mL)*	54.2 [35.1; 84.6]	72.1 [62.1; 95.6]	**0.05**
**25OHD (ng/ml)^a^**	24.5 [18.1; 29.6]	22.8 [14.5; 33.7]	0.98
**Thyroid function and immunity**			
* - TSH (mIU/l)*	1.12 [0.735; 1.73]	1.51 [0.970; 2.00]	0.3
* - fT3 (pmol/l)*	3.59 [3.00; 4.2]	3.75 [3.30; 4.17]	0.91
* - fT4 (ng/dl)*	1.09 [1.04; 1.18]	1.15 [1.05; 1.22]	0.42
* - Anti-TPO antibodies^b^*	3.3%	17%	0.19
* - Anti-TG antibodies* **^c^**	0%	17%	0.08

The data are expressed as the median [interquartile range] or the frequency (percentage).

GH, growth hormone; IGF1, insulin-like growth factor 1; 25OHD, 25-hydroxy vitamin D; TSH, thyroid-stimulating hormone; fT3, free triiodothyronine; fT4, free thyroxine; TPO, thyroperoxidase; TG, thyroglobulin.

Missing data: ^a^n=7, ^b^n=17, ^c^n=18.

## Discussion

We have studied hormonal levels in FF during IVF and their association with oocytes quality. Our results show that levels of GH and IGF1 were higher in the normal oocyte cohort than in the abnormal oocyte cohort. At the opposite, there was no association with follicular levels of TSH, fT3, fT4, antithyroid antibodies, or 25OHD.

In our study, we found that the fertilisation rate was significatively lower in the abnormal cohort group, as also demonstrated by Setti et al.’s meta-analysis (19) and discussed recently by Camargos et al. (20). As was also the case in Setti et al.’s meta-analysis, we did not observe a significant difference in markers of embryonic development. It is noteworthy that the literature data on other markers of oocyte quality impact are inconsistent. Some researchers reported poor cleavage rates (21, 22), embryonic development rates (23) and blastulation rates (24) with poor-quality embryos (21, 25). We found much the same pregnancy and live birth rates as in some studies (3, 26–28) but not in others (29, 30). These contrasting results for ICSI outcomes might be due to interstudy differences in the assessment of oocyte abnormalities and/or the impact of spermatozoid quality on embryo development.

We found that 59% of the oocytes presented at least one morphological abnormality. This is in line with literature reports on COS for IVF, in which the proportion of abnormal oocytes ranges from 10% to 64% (1–5). Our data for the proportions of oocytes with one abnormality or two or more abnormalities were within the ranges reported in the literature (41%-63%, and 15%-32%, respectively) (3, 21, 31, 32). We found that proportion of oocytes with two or more abnormalities was significantly greater in the abnormal oocyte cohort than in the normal oocyte cohort. The two cohorts were similar with regard to the distribution of the types of oocyte morphological abnormality. Cytoplasmic granulation accounted for over half of the morphological abnormalities; this proportion is higher than those reported in the literature, ranging from 5.4% and 24% (22, 32, 33). This high proportion in both cohort may be due to environmental factors as we already discussed in a previous study (34).

In our study, we observed an association between higher follicular levels of GH and IGF1 and oocyte quality. To the best of our knowledge, the correlation between follicular GH and IGF1 levels and oocyte cohort morphology has not previously been evaluated in IVF. Follicular GH and IGF1 levels appear to be associated with oocyte maturation: one study found a positive correlation between follicular GH and the number of oocytes collected (35) and another study found a positive correlation between follicular IGF1 and the number of mature oocytes (36). However, these associations were not found in other studies (37, 38). It has also been demonstrated that follicular GH and IGF1 levels are higher in follicles containing mature oocytes than in follicles containing atretic oocytes (39), as the density of IGF receptors in granulosa cells (40). With regard to other IVF parameters, some studies (41, 42) but not others (38, 43) have found that follicular GH and IGF1 levels were positively correlated with the fertilisation rate. Overall, higher follicular GH and IGF1 levels appear to be associated with better oocyte competency in IVF. This hypothesis is also supported by the beneficial effect of adjuvant GH treatment on IVF outcomes. Indeed, many studies of GH treatment have reported a better ovarian response to stimulation, greater numbers of oocytes and embryos, and higher pregnancy and live birth rates in poor responders (44). Furthermore, during *in vitro* maturation of human oocytes, the addition of IGF1 to the culture medium is associated with a greater number of mature oocytes (45, 46) and a lower proportion of oocytes with morphological abnormalities (46). However, the association that we highlight does not allow us to conclude whether lower levels of IGF1 would be a cause or a consequence of a poorer oocyte quality. Current knowledge on the role of somatotropic axis on ovarian function reinforce the hypothesis of the beneficial effect of GH and IGF1 on oocyte competency. In humans, GH is involved in (i) initiating and sustaining the development of primordial follicles into preovulatory human follicles, (ii) cytoplasmic and nuclear maturation in the oocyte, and (iii) cumulus cell expansion (47, 48). In addition to these direct effects on oocyte development, adjuvant treatment with GH in IVF is associated with lower FF levels of oxidative stress markers (49). Furthermore, systemic and/or local IGF1 stimulates the proliferation, differentiation and activity of granulosa cells (48). GH and IGF1 are also synergic with gonadotropins, as they improve the ovary’s responsiveness to FSH and LH by increasing expression levels of the cognate receptors (50, 51). Our design study using a pool of FF for each patient does not allow us to extrapolate the pathophysiological impact of the somatotropic axis on FF composition and oocyte competency for each individual follicle.

Given that the vitamin D receptor is detected in stromal, granulosa and thecal ovarian cells (52, 53) and that the concentration of 25OHD in FF is positively correlated with the serum (54), we hypothesised that the FF 25OHD level would be associated with oocyte cohort quality. However, we did not find any evidence of such a relationship. Although the possible association with oocyte morphology has not previously been assessed, other IVF parameters have been evaluated in the literature; there was no difference in the number of oocytes collected (55, 56) or in the oocyte maturation rate as a function of the follicular 25OHD level (56). Conversely, some researchers have observed a negative correlation between the follicular 25OHD level and the fertilisation rate (57). It is noteworthy that supplementation with the active form of vitamin D (i.e. 1-25OHD) increases granulosa cell proliferation and differentiation and oocyte maturation (13, 14).

In our study population of euthyroid women, the follicular TSH, fT3, fT4 and antithyroid antibody levels were similar in the normal and abnormal oocyte cohorts. In the literature, only Cai et al. studied the link between follicular levels of TSH/TH and IVF parameters. They only observed a positive correlation between follicular TSH level and embryo quality. However, oocyte morphology was not assessed in this study (58). The fact that TSH receptors are expressed throughout folliculogenesis (59) suggests that this hormone has a major role in oocyte development independently of THs, since the latter are not synthesised locally. fT3 improves granulosa cell proliferation and differentiation (9), and THs inhibit granulosa cell apoptosis (10). The positive correlation between follicular and serum hormone levels suggests the presence of passive transport into the follicular compartment (58). However, since human granulosa cells and the superficial epithelial cells of the ovary have been shown to express deiodases 2 and 3 (59, 60), the correlation might indicate the conversion of T4 to active T3 or inactive T3r. This conversion might be influenced by follicular action TSH on deiodases.

Antithyroid antibodies are detected in about 10% of women of childbearing age; we found a similar proportion in the present study. The difference in the prevalence of antithyroid antibodies between the abnormal and normal oocyte cohorts was not statistically significant. This finding is in line with most of the literature data; in general, no differences were observed in terms of the number of oocytes collected (61) or [with the exception of one study (62)] the fertilisation rate (58, 61). The follicular and serum concentration levels of antithyroid antibodies are positively correlated (58); this is suggestive of passive transport, and so the antithyroid antibodies present in the FF might simply be “bystanders”.

One of the limitations of our study is a small population. It would be valuable to confirm our present findings (i.e. an association between follicular GH and IGF1 levels and oocyte cohort quality) in a larger cohort. Studying each follicle individually could also be an important point to investigate relationships between hormone concentrations and each type of oocyte morphological abnormality. Furthermore, the assessment of oocyte morphological abnormalities in women with hormonal deficit might be informative. Indeed, we have observed a dramatic reduction in the number of oocyte morphological abnormalities after GH replacement therapy in patient with GH deficiency (63). However, this type of study would not be ethically possible in women with hypothyroidism because the establishment of euthyroidism in pregnancy is clinically necessary. Experiments in animal models might be informative.

## Conclusion

This pilot cohort study is the first who evaluated the association between FF hormone concentrations and oocyte cohort quality. Follicular levels of GH and IGF1 were significantly higher in a normal oocyte cohort than in an abnormal oocyte cohort. However, we did not observe an association with follicular levels of TSH, fT3, fT4, 25OHD, or antithyroid antibodies. The GH/IGF1 system appears to be important for oocyte development and competency. Further characterisation of these hormones’ actions is now necessary.

## Data Availability Statement

The original contributions presented in the study are included in the article/supplementary material. Further inquiries can be directed to the corresponding author.

## Ethics Statement

The studies involving human participants were reviewed and approved by CPP Amiens, France (reference: RCB 2011-A00634-37). The patients/participants provided their written informed consent to participate in this study.

## Author Contributions

FS, RC, AD, and RD contributed to conception and design of the study. MS carried out the biological analyzes of follicular fluid. DB and EL carried out the morphological evaluation of oocytes. MK allowed proofreading of English. FS wrote the first draft of the manuscript. FS, MS, DB, and EL wrote sections of the manuscript. All authors contributed to manuscript revision, read, and approved the submitted version.

## Conflict of Interest

The authors declare that the research was conducted in the absence of any commercial or financial relationships that could be construed as a potential conflict of interest.

## Publisher’s Note

All claims expressed in this article are solely those of the authors and do not necessarily represent those of their affiliated organizations, or those of the publisher, the editors and the reviewers. Any product that may be evaluated in this article, or claim that may be made by its manufacturer, is not guaranteed or endorsed by the publisher.
